# Society and Its Lunatics

**Published:** 1856-03

**Authors:** 


*Society and its Lunatics.*—There are at the present moment hundreds, if not thousands, of people in this country, and at large, who are, at least, the subjects of delusions, if not decidedly lunatic. This may at first sight appear a startling statement, but it is nevertheless true, with all its hideousness. According to the census of 1851, there were, in Lunatic Asylums in Great Britain, 18,800 Lunatics,* which gives a proportion to the general population of 1 in 1115. But the census for Great Britain gives no particulars as to the number of Lunatics or Idiots *at large* in 1851; so that we have no direct means of coming to a conclusion upon the subject. The Census of Ireland, however, ("Report on the Status of Disease") throws in some light here, from which certain general deductions may be drawn. According to this document, there were in Ireland, in 1851, 4635 Lunatics and Idiots at large, giving a proportion of one such to every 1413 of the population; and, from the difficulty and delicacy of the task of getting such information, we may, at least, consider that the number is not overstated. Adopting, then, this proportion for Great Britain, it may be pretty safely asserted that there are now abroad, under no restraint, except such as their own friends can or may impose, no less than 15,000 Lunatics and Idiots,—many apparently harmless, no doubt; others requiring but little to inflame the morbid passion; and others, again, heaping up their wrath for a fitting opportunity. This is a state of things to which civilized society ought not to be exposed. The general population has a right to be protected from those raving madmen, monomaniacs, and delusionists. Here are our incipient maniac murderers; and yet society takes no cognizance of them, till, it may be, a wretched mother or father stands at the felon's bar, with hands imbrued in the blood of his or her offspring. Then, when the mischief is done, the recollection of all the family and friends is ransacked to get up evidence as to insanity; and the victims of a delusion being already sacrificed, the maniac is *then* committed to safe custody at the expense of the State. Now, there appears to us a growing danger in this state of things. It is well known that there is great diversity of opinion as to the desirableness or policy of capital punishment, and petty jurymen are but men. Twelve men may be perfectly honest, and hold sacred the oath they have taken; but upon them rests the fearful responsibility of sending a fellow-creature to the gallows. Evidence

* This number does not include the lunatics in work-houses.

is adduced before them of acts not consistent with a well-regulated mind; the circumstances are coloured by the prisoner's counsel; and we say it is not at all surprising that, acquittal being out of the question, the jury should lean to the merciful alternative, especially as they know it will secure society from further mischief. Hence arises another plea in the mind of the would-be (but not lunatic) murderer; and in proportion to the success of such pleas, will be the danger to society.

The present period has been peculiarly rife in murders; and, with hardly any exceptions, the broad statement is made at the outset of proceedings—as in the case of Bousfield, who this week murdered his wife and three children—"for some time past his conduct has been very eccentric;" or, as in that of the child murder at Sheffield, "it appears that he has been subject to occasional fits of insanity for some time past;" and many other such cases will occur to our readers. Now, is there no remedy for such a condition of things as this? We think there is. Say that there are 15,000 persons abroad in Great Britain certainly demented, and of whom none can prophesy harmlessness. Why should not the evidence as to insanity be available to the State before murder or mischief is committed, as it is available for the prisoner after he has committed murder?

How is this to be secured? We say, pass an enactment that, in no case of murder shall evidence of *previous* insanity be admissible in answer to a charge of murder, unless evidence as to the condition of the lunatic, or as to hereditary mental disorder in the family, has been previously sworn to before a magistrate, justice of the peace, or other such officer; and let it be the duty of the State to provide such security for the public, as each case, after close Professional examination, may call for. Apply such a scheme to the numerous cases which have lately been before the public, and we have little doubt that in all cases of real insanity the lives of the victims would have been saved, no wrong would be done to the demented, and a stop would be effectually put to the trumping up of false pleas of insanity, while some check would be interposed to the propagating of mental disorder by marriage, over which now there is no control. In reference to this latter point we may state that, by the Census of 1851, it was found that in Ireland there were 647 married males, and 1074 married females, lunatics, about a ninth part of whom were at large, and that more than one half of all the lunatics and idiots were at the procreating period of life.—*Medical Times and Gazette.*

